# Otogenic temporomandibular septic arthritis in a child: a case report and a review of the literature

**DOI:** 10.1186/s13052-019-0682-2

**Published:** 2019-07-22

**Authors:** Massimo Luca Castellazzi, Laura Senatore, Giada Di Pietro, Raffaella Pinzani, Sara Torretta, Ilaria Coro, Antonio Russillo, Irene Borzani, Samantha Bosis, Paola Marchisio

**Affiliations:** 1 ASST NORDMILANO, Sesto San Giovanni Hospital, Paediatric and Neonatology Unit, Sesto San Giovanni, Milan, Italy; 2 Fondazione IRCCS Ca’ Granda Ospedale Maggiore Policlinico, Paediatric Highly Intensive Care Unit and Department of Pathophysiology and Transplantation, University of Milan, Milan, Italy; 3 Fondazione IRCCS Ca’ Granda Ospedale Maggiore Policlinico, Paediatric Highly Intensive Care Unit, Milan, Italy; 4 Fondazione IRCCS Ca’ Granda Ospedale Maggiore Policlinico, ENT Unit, Department of Clinical Sciences and Community Health, University of Milan, Milan, Italy; 5 Fondazione IRCCS Cà Granda Ospedale Maggiore Policlinico, Maxillofacial and Dental Unit, Milan, Italy; 6 Fondazione IRCCS Ca’ Granda Ospedale Maggiore Policlinico, Radiology Unit - Paediatric Division, Milan, Italy

**Keywords:** Temporomandibular joint, Septic arthritis, Acute otitis media, Children

## Abstract

**Background:**

Acute otitis media is one of the most common infectious diseases in the paediatric age and although its complications such as acute mastoiditis have become rare thanks to improvements in therapeutic approaches, possible serious complications such as septic arthritis of the temporomandibular joint may develop. A prompt diagnosis and adequate treatment are essential to achieving the best outcome and avoiding serious sequelae. We describe a case occurring in a previously healthy 6-year-old female and review the literature currently available on this topic.

**Case presentation:**

The patient presented a right temporomandibular septic arthritis with initial mandibular bone involvement secondary to acute otitis media. She presented with torcicollis, trismus, right preauricular swelling over the temporomandibular joint and was successfully treated with antibiotic treatment alone.

**Conclusions:**

Septic arthritis of the temporomandibular joint is a rare complication of acute otitis media or acute mastoiditis in children. It should be suspected in patients presenting with trismus, preauricular swelling or fever. No guidelines on the diagnosis and treatment of this infectious disease are currently available.

## Background

More than 80% of children under 3 years of age experiences at least one episode of acute otitis media (AOM) and about one third of children has a recurrence by the age of 3 years, making AOM one of the most common paediatric infectious disease [1, 2]. Moreover, in Italy it has been shown that the incidence of AOM in children under 5 years of age in outpatient care is around 16% [3]. One of the most important complications of AOM in the paediatric age is mastoiditis [4].

Other severe complications include subperiosteal abscess, facial paralysis, serous and suppurative labyrinthitis, sigmoid sinus thrombosis, epidural or intracerebral abscess, meningitis, petrous apicitis, and otitic hydrocephalus. Fortunately, modern treatment options make these complications rare in developed countries [5].

Septic arthritis of the temporomandibular joint (TMJ) is a rare complication of AOM with or without otomastoiditis in children; however, a prompt diagnosis and appropriate treatment are crucial to reducing the risk of complications such as TMJ ankylosis and achieving the best outcome [6]. There are currently no guidelines for the diagnosis and treatment of TMJ septic arthritis.

Here we report the case of a previously healthy child with right TMJ septic arthritis secondary to AOM. We also review literature reports of otogenic cases of TMJ septic arthritis in children, published in English since 2000, in order to discuss the main clinical findings and the diagnostic and therapeutic strategies for this condition.

## Case presentation

**Fig. 1:**
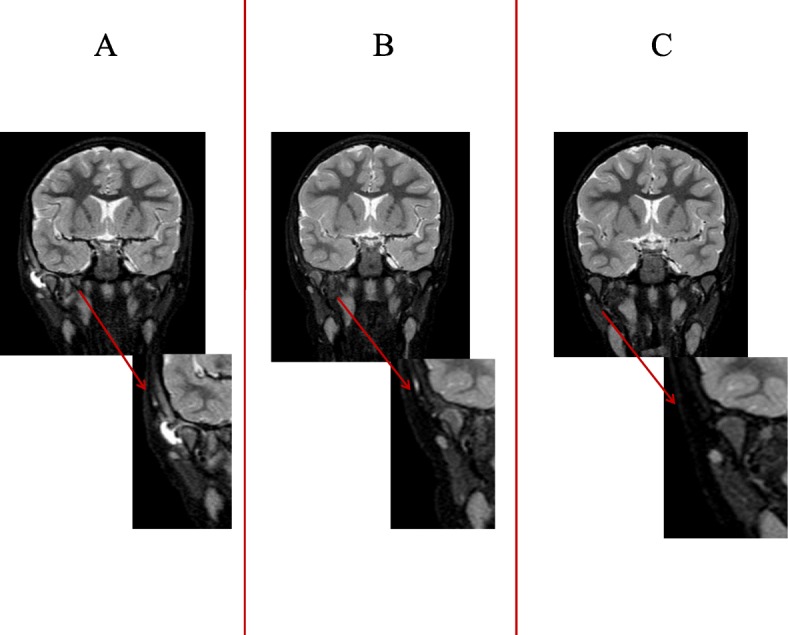
**a** STIR coronal image performed in the acute phase shows hyperintensity of the right mandibular condyle consistent with bone oedema/inflammation and minimal effusion in the articular space, suggesting osteoarthritis. **b** STIR coronal image performed 1 month after discharge shows a reduction in bone hyperintensity and complete reabsorption of the effusion. **c** STIR coronal image performed 10 months later shows complete recovery of the normal bone intensity of the right mandibular condyle

A previously healthy and fully immunised six-year-old girl was admitted to our hospital with right otalgia, progressive trismus and right neck pain for the previous 3 days. She had been on oral antibiotic treatment for right AOM with amoxicillin-clavulanate (80 mg/kg/day) for 2 days without improvement. There was no history of trauma. On admission, the child was in good general conditions and presented with torcicollis, trismus, right preauricular swelling over the temporomandibular joint. On otoscopy right tympanic hyperemia with the evidence of middle ear fluid consistent with AOM was observed. Significant pain on chewing and right-sided cervical lymphadenopathy were also reported. She was apyretic and the rest of her clinical examination was normal. An ultrasound scan revealed a fluid collection of 4 mm in size in the right TMJ. A contrast-enhanced computed tomography (CT) scan of the head and neck confirmed the presence of effusion in the right TMJ, consistent with the diagnosis of septic arthritis, with surrounding lymphadenopathy and initial signs of bone rarefaction of the right mandibular condyle. An opacity of the right mastoid was detected radiologically, but there were no clinical signs of mastoiditis. These findings were also confirmed by the subsequent magnetic resonance imaging (MRI) of the head and neck (Fig. 1a). On admission, laboratory tests showed a white blood cell count of 23,420/mm^3^ with neutrophil predominance and elevated C-reactive protein (CRP) of 8.1 mg/dL (normal value < 0.5 mg/dL). A blood culture was also performed but resulted negative. On the basis of these findings, broad spectrum intravenous antibiotic treatment with ceftriaxone (75 mg/kg/day) was introduced, but 3 days later it was replaced with a combination of piperacillin-tazobactam (100 mg/kg/day) and vancomycin (40 mg/kg/day) due to minimal clinical improvement. This antibiotic regimen was well tolerated by the patient and it was continued for a total of 4 weeks with a gradual resolution of the symptoms and normalisation of the white blood cell count and CRP. The patient was discharged in good general conditions under an oral antibiotic treatment with clindamycin (20 mg/kg/day) for 2 weeks. The MRI of the head and neck performed 1 month later revealed a further improvement in joint effusion and a reduction in bone involvement (Fig. 1b); ten months after onset a complete resolution was observed on MRI (Fig. 1c).

## Discussion and conclusion

Septic arthritis of TMJ is a rare complication of AOM with or without otomastoiditis in children. We identified 34 cases of paediatric TMJ septic arthritis published since 2000 [6–14]. Of these, 5 were excluded because the source of the TMJ septic arthritis was not of otogenic origin or was not specified [7, 13, 14]. 8 were case reports, in which 6 cases of TMJ septic arthritis were secondary to AOM, [6–13]. Luscan et al. in a prospective study from 2014 to 2015 observed that of 45 patients with acute otomastoiditis, 15 had a TMJ effusion [15]. Finally, in a recent multicentre retrospective study by Burges et al., 9 cases of paediatric TMJ septic arthritis were described. Of these, in 7 cases (78%) the primary middle ear infection was acute mastoiditis, whereas in the two remaining cases the origin of TMJ septic arthritis was AOM [16]. Table 1 summarises the main findings of these reports.

**Table 1:** Principal characteristics of previously reports of paediatric septic arthritis of the temporomandibular joint

Author, year of publication	Kind of study	Age & Sex	Initial site of infection	Initial sign of TMJ infection	Imaging	Surgical treatment	Antibiotic treatment	Bacteria involved	Outcome
Hadlock TA et al., 2001 [8]	Case report	11 y, F	AOM and mastoiditis	Otalgia, trismus, left-side neck tenderness	CT scan	Mastoidectomy, myringotomy, aspiration of the TMJ	Broad spectrum intravenous antibiotics, followed by oral antibiotics. Not specified the kind of antibiotic	Group A *Streptococcus*	None
Amos MJ et al., 2007 [9]	Case report	6 y, F	Suspected AOM	Otalgia, facial swelling, fever, jaw pain	Orthopantomogram; Ultrasound scan	Aspiration of the TMJ	Flucloxacillin and metronidazole	Unknown	None
Gayle EA et al., 2013 [10]	Case report	6 y, M	AOM	Fever, otalgia, rhinorrhoea, vomiting, trismus	CT scan	Arthrocentesis	Intravenous ampicillin/sulbactam followed by oral amoxicillin/clavulanate	Group A *Streptococcus*	None
Bast F et al., 2015 [6]	Case report	7 y, M	AOM	Pain, swelling, trismus	MRI	2 aspiration and washout of the TMJ	Intravenous ceftriaxone	Group A *Streptococcus*	None
Tsai C et al., 2017 [11]	Case report	5 y, M	AOM	Fever, otalgia, swelling, trismus	CT scan (that revealed concomitant Luc’s abscess)	TMJ arthrotomy	Intravenous ceftriaxone followed by oral amoxicillin/clavulanate	Group A *Streptococcus*	None
Dubron K et al., 2017 [12]	Case report	7 y, M	AOM	Otalgia, trismus	CT scan	Arthrocentesis	Intravenous amoxicillin/clavulanate	Group A *Streptococcus, Staphylococcus epidermidis*	None
Luscan et al., 2016 [15]	Prospective	15 of the 45 patients enrolled	Otomastoiditis	Unknown	CT scan	Not available	Unknown	Unknown	2 patients presented TMJ ankylosis
Burgess et al., 2017 [16]	Retrospective	9 patients, 6 M, mean age 2.1 y	Acute mastoiditis in 7 cases, AOM in 2 cases	Preauricular swelling (5 cases), trismus (1 case)	CT scan	Surgical drainage, mastoidectomy	Antibiotic treatment (not clarify)	*Fusubacterium necrophorum* (3 cases)	Long-term ankylosis in 6 cases

The clinical presentations of TMJ septic arthritis include fever, trismus, preauricular swelling, and TMJ tenderness [13].

In our case, there was no history of fever, but the other main clinical signs were all present.

Although a diagnosis of TMJ septic arthritis can be suspected on the basis of clinical findings, radiological confirmation is essential in order to identify other possible complications. Contrast-enhanced CT is the most frequent imaging technique used in the assessment of TMJ [7, 8, 10–13]. CT is also easy to perform in children and provides a better distinction between bone and soft tissues [16].

In the case described by Bast et al., MRI was used for diagnosis, whereas Amos et al., in emergent evaluation settings, based their diagnosis on orthopantomography and ultrasound scans [6, 9].

In our case, the ultrasound scan revealed the presence of a fluid collection in the right TMJ, but the CT scan of the head and neck was essential to describing the initial bone involvement. MRI was useful for monitoring the radiological evolution of the condition and preventing our patient from being exposed to radiation.

Laboratory findings often reveal an increase in the white blood cell count and CRP but their specificity and sensitivity are too poor to allow diagnosis without further clinical and radiological data [13]. The most frequent causative organism identified is group A *Streptococcus*. In one case, it was associated with *Staphylococcus epidermidis*. In the retrospective study conducted by Burgess et al., of 9 children with TMJ septic arthritis only 3 had an identified causative organism and it was *Fusobacterium necrophorum* [16]. It is interesting to note that as conventional methods of culturing may not be sensitive enough for the identification of the causative pathogen, Polymerase Chain Reaction was recently described as a useful test for identifying the pathogen [6, 12].

No guidelines on the treatment of TMJ septic arthritis are currently available. Broad spectrum intravenous antibiotic treatment is essential and can be modified on the basis of the antibiograms obtained from the cultural investigations [17]. The duration of the antibiotic treatment is still a matter of debate. In our case, we opted for a 4-week course of intravenous antibiotic followed by 2 weeks of oral antibiotic due to initial bone involvement.

Alongside antibiotics, surgery plays a key role in the treatment of septic arthritis in children [18]. Different surgical options are available and these include needle aspiration, aspiration with arthroscopic joint lavage and arthrotomy. However, inadequate evidence is available on what the best surgical treatment option is. Furthermore, surgery should not be considered in patients who respond to ongoing antibiotic treatment, as in our case [17]. Joint aspiration could be useful for decompression, elimination of inflammatory contents, and to facilitate synovial fluid analysis to guide antibiotic therapy, especially in the more severe cases.

Currently, there is no specific data on mandibular physiotherapy; however, different reports have suggested that it could be useful in order to improve condyle excursion and prevent fibrotic adhesions. Active opening and bite exercises in particular could improve maximal incisal opening [13].

To conclude, septic arthritis of the TMJ is a rare but potentially severe complication of AOM or otomastoiditis in children. It should be suspected in the presence of trismus, jaw pain and preauricular swelling, in order to perform radiological examinations to confirm the diagnosis and to initiate adequate antibiotic and, if necessary, surgical treatment. A prompt diagnosis and adequate treatment are essential if severe complications as destruction of the synovial tissues, fibrotic adhesions and joint ankylosis are to be avoided [9].

## Data Availability

Data sharing was not applicable to this case report because no datasets were generated or analysed during the study.

## Abbreviations

AOM: Acute otitis media
CRP: C-reactive protein
CT: Computed tomography
MRI: Magnetic resonance imaging
TMJ: Temporomandibular joint
